# Role of the C5a-C5a receptor axis in the inflammatory responses of the lungs after experimental polytrauma and hemorrhagic shock

**DOI:** 10.1038/s41598-020-79607-1

**Published:** 2021-01-25

**Authors:** Shinjini Chakraborty, Veronika Eva Winkelmann, Sonja Braumüller, Annette Palmer, Anke Schultze, Bettina Klohs, Anita Ignatius, Axel Vater, Michael Fauler, Manfred Frick, Markus Huber-Lang

**Affiliations:** 1 Institute of Clinical and Experimental Trauma-Immunology, Ulm University Medical Center, Helmholtzstrasse 8/1, 89081 Ulm, Germany; 2 Institute of General Physiology, Ulm University, Albert-Einstein-Allee 11, 89081 Ulm, Germany; 3 Institute of Orthopedic Research and Biomechanics, Ulm University Medical Center, Helmholtzstrasse 14, 89081 Ulm, Germany; 4 Aptarion Biotech AG, Max-Dohrn-Str. 8-10, 10589 Berlin, Germany

**Keywords:** Immunology, Physiology, Diseases, Molecular medicine

## Abstract

Singular blockade of C5a in experimental models of sepsis is known to confer protection by rescuing lethality and decreasing pro-inflammatory responses. However, the role of inhibiting C5a has not been evaluated in the context of sterile systemic inflammatory responses, like polytrauma and hemorrhagic shock (PT + HS). In our presented study, a novel and highly specific C5a L-aptamer, NoxD21, was used to block C5a activity in an experimental murine model of PT + HS. The aim of the study was to assess early modulation of inflammatory responses and lung damage 4 h after PT + HS induction. NoxD21-treated PT + HS mice displayed greater polymorphonuclear cell recruitment in the lung, increased pro-inflammatory cytokine levels in the bronchoalveolar lavage fluids (BALF) and reduced myeloperoxidase levels within the lung tissue. An in vitro model of the alveolar-capillary barrier was established to confirm these in vivo observations. Treatment with a polytrauma cocktail induced barrier damage only after 16 h, and NoxD21 treatment in vitro did not rescue this effect. Furthermore, to test the exact role of both the cognate receptors of C5a (C5aR1 and C5aR2), experimental PT + HS was induced in C5aR1 knockout (C5aR1 KO) and C5aR2 KO mice. Following 4 h of PT + HS, C5aR2 KO mice had significantly reduced IL-6 and IL-17 levels in the BALF without significant lung damage, and both, C5aR1 KO and C5aR2 KO PT + HS animals displayed reduced MPO levels within the lungs. In conclusion, the C5aR2 could be a putative driver of early local inflammatory responses in the lung after PT + HS.

## Introduction

Polytrauma is a life-threatening multiple injury which represents a major global as well as clinical burden and is a common cause of mortality among younger people^1^. It is a form of sterile systemic inflammatory response syndrome (SIRS), which damages organ structures, tissue integrity and cells, resulting in the release of danger-associated molecular patterns (DAMPs)^2^. At the early stages of polytrauma, the recognition of DAMPs is mediated through the activation of the complement cascades^3,4^, and a robust inflammatory response is generated through the recruitment of leukocytes and a cytokine storm. Frequently termed as complementopathy, it is characterized by a rapid and robust consumption of the complement, together with the generation of the anaphylatoxins of the complement pathways, C3a and C5a, from factors C3 and C5 respectively^5,6^. Acute coagulopathy is also a common occurrence in polytrauma patients^7^, whereby the activation of coagulation cascades may putatively also contribute to the activation of the complement cascades^8,9^. These potent mediators of inflammation in turn promote a complex sequela of events, establishing an elaborate immunopathology after trauma. The generated anaphylatoxins C3a and C5a extend their functionality through various mechanisms. For example, in addition to recruiting neutrophils, C5a acts as an immuno-metabolic “switch”, which can alter the microenvironment to induce acidosis^10^. Such effects exerted by the anaphylatoxins not only shape an intricate immune response, but also form the basis of organ dysfunction. Considered as a major driver of organ barrier damage, hemorrhagic shock facilitates the development of multiple organ dysfunction (MODS) following experimental and clinical polytrauma^2,11,12^, and complement is actively involved in its respective pathophysiology^13^. During SIRS, the lung is one of the major target organs, where disruption of the alveolar-capillary barrier is known to contribute to the development of acute respiratory distress syndrome (ARDS)^14^. Moreover, mortality from ARDS has been shown to correlate with polymorphonuclear cell (PMN) recruitment within the lung^15^. Existing literature has also described that in response to DAMPs, the alveolar space contains high concentrations of C5a after lung injury^16–18^, and, therefore, could benefit the most from a C5a blockade to inhibit the recruitment of inflammatory cells within the alveoli^18,19^.

C5a may cause infectious complications, including sepsis and sepsis-induced multiple-organ failure^20^. It can bind to two receptors—C5aR1 and C5aR2, both G-protein coupled receptors, whose effect has been tested in various models of infection and inflammation^21,22^. Several inhibitory strategies have targeted C5 to alter the consequences of local and systemic complement activation in other diseases^23,24^. In the context of SIRS, inhibiting C5 cleavage in baboons with *Escherichia coli*-induced sepsis with a macrocylcic compound RA101295 induced a prompt effect in reducing proinflammatory responses as early as 2 h post sepsis induction in terms of reduced circulatory levels of C3b, C5a and soluble C5b–9 and attenuated damage of the lung and kidney^25^. Recently, a novel C5a-neutralizing aptamer was proven to effectively abrogate C5a-mediated inflammatory responses and MODS in an experimental model of sepsis^26^, where it was shown to reduce inflammatory cytokines in plasma and peritoneal lavage fluid, reduce organ damage and improve survival. Moreover, blocking the terminal C5b–9 complex has also helped in resolving the resultant damage response in a non-human primate model of trauma-hemorrhagic shock^13^. Effector components generated during complement pathway activation are putative candidates to test whether their respective blockade can rescue SIRS-associated immune and organ dysfunctions. These may include the potent anaphylatoxin C5a, where blocking C5a–C5aR interaction could prove to be particularly relevant in this regard. Such approaches have even shown promising results with overall improved leukocyte function, organ function and clinical outcome in experimental sepsis-induced MODS^27^. An absence of C5aR1 or C5aR2 has been described previously to attenuate C5a-mediated effects in septic conditions^28,29^. In a murine model of lipopolysaccharide (LPS)-challenged acute lung injury, C5aR1 activity, in the absence of C5aR2, enhanced pro-inflammatory cytokine levels, myeloperoxidase (MPO) levels and bronchoalveolar lavage fluid (BALF) cell count^30^. As opposed to chronic inflammatory pathologies of the lung or acute lung injury induced by infection, a direct physical damage to the lung may entail damage responses which may be different. However, to our knowledge, there are neither clinical nor preclinical experimental studies to confirm any benefit of C5a-inhibition in the early phase after PT + HS, particularly in the context of protecting against lung inflammation and damage. An effective neutralization of C5a may even exhibit harmful effects, because some important functions of neutrophils to clear tissue debris and microbes may be compromised. In the present study, we have, therefore, examined an effective C5a inhibition strategy using intravenous injection of a C5a-neutralizing L-aptamer^31^ in a highly standardized murine model of PT + HS. A novel alveolar-capillary barrier model was used to investigate the alveolar-capillary barrier in detail, and the effects of C5a-blockade in vitro. Additionally, the specific contribution of C5a signaling cascades during PT + HS was investigated using C5a-receptor knockout mice.

## Results

### In vitro effect of C5a L-aptamer treatment on C5a-induced chemotaxis of PMNs and in vivo effect in murine PT + HS

**Figure 1:**
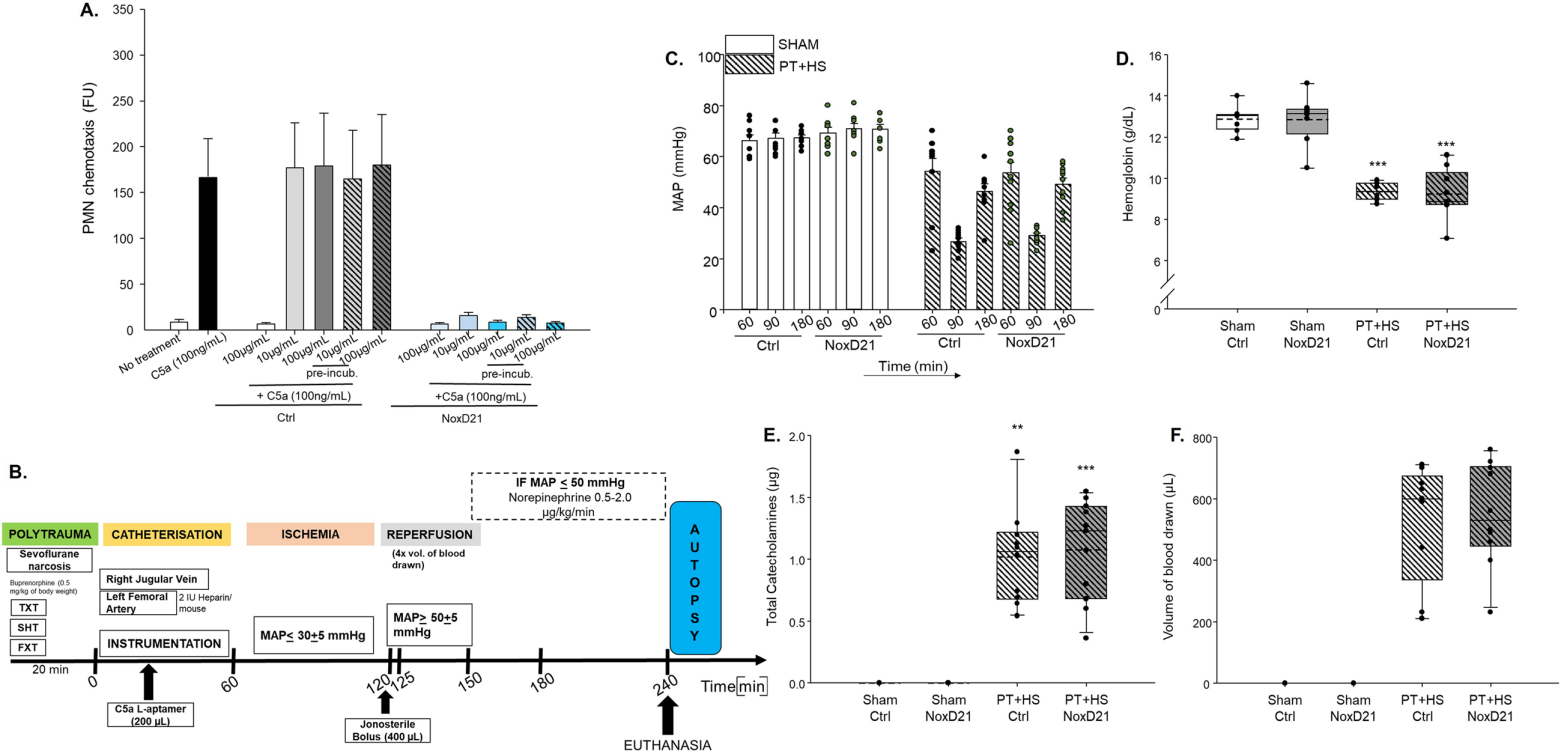
C5a inhibition with L-aptamer diminishes chemotaxis of PMNs in vitro but does not affect the vital characteristics in a PT + HS mouse model. (**A**) NoxD21 and a control (Ctrl; reversed NoxD21 or rev NoxD21) were used to assess their effects on PMN chemotaxis. FU stands for fluorescence units. (**B**) Schematic diagram of the PT + HS model. (**C**) MAP measurements, (**D**) hemoglobin concentration, (**E**) volume of blood drawn to induce HS in PT + HS animals and (**F**) total catecholamine required post-resuscitation are shown for the sham and PT + HS groups. For (**C**) and (**D**), two-way ANOVA was performed with Student-Newman-Keul’s post-hoc analysis, where the ** and *** indicate p < 0.01 and p < 0.001, respectively, for comparison of each PT + HS group with its corresponding sham group. For (**E**) and (**F**) Kruskal–Wallis test with Dunn’s post-hoc analysis was performed, where ** and *** indicate p < 0.01 and p < 0.001, respectively, for comparison between sham groups and PT + HS groups. Animals per group for all measurements were n = 8–11.

NoxD21 is a polyethylene glycol PEGylated L-aptamer which can bind to both C5 and C5a, thus exerting an effective blockade of C5a^31^. This L-aptamer consists of a modification with a 40 kDa PEG, which confers it an increased circulatory half-life. Firstly, an in vitro experiment was performed to determine the efficacy of NoxD21 in inhibiting PMN chemotaxis (Fig. 1A). The control (Ctrl) used was a PEGylated reversed aptamer revNOX-D21 (referred to as Ctrl throughout) that does not bind to C5a. Two concentrations of NOX-D21 and control were tested (10 μg/mL and 100 μg/mL corresponding to 0.76 µmol/L and 7.6 µmol/L, respectively), with or without C5a (100 ng/mL) incubation. C5a L-aptamer significantly diminished chemotaxis in PMNs when co-incubated with C5a, whereas the scrambled L-aptamer displayed no such inhibition (Fig. 1A).

Subsequently, we performed in vivo experiments emulating PT + HS^11^ in mice to investigate the aptamer’s effect on inflammatory responses and organ function. Because NoxD21 was capable of effectively blocking the functional effects of C5a in vitro, we applied this blocking strategy for the systemic inflammatory responses early after PT, which to date have been unexplored. An experiment was designed, designating 8–12 mice in each of the four groups: Sham (Ctrl treated), Sham (NoxD21 treated), PT + HS (Ctrl treated) and PT + HS (NoxD21 treated). All animals were instrumented and observed as described previously^8^, in the materials and methods and in Fig. 1B. Sham animals were anesthetized, and arterial and venous catheterization were placed without inducing PT or ischemia. It was observed that fluctuation of the mean arterial pressure (MAP) after HS induction (Fig. 1C), hemoglobin concentration in blood post sacrifice (Fig. 1D), volume of blood drawn to induce HS (Fig. 1E) and the total catecholamine requirement (Fig. 1F) did not differ between the Ctrl-treated and NoxD21-treated PT + HS animal groups. This indicated that the PT + HS was uniformly performed in the animals and the vital statuses were unaffected by the blockade of C5a with NoxD21.

**Figure 2:**
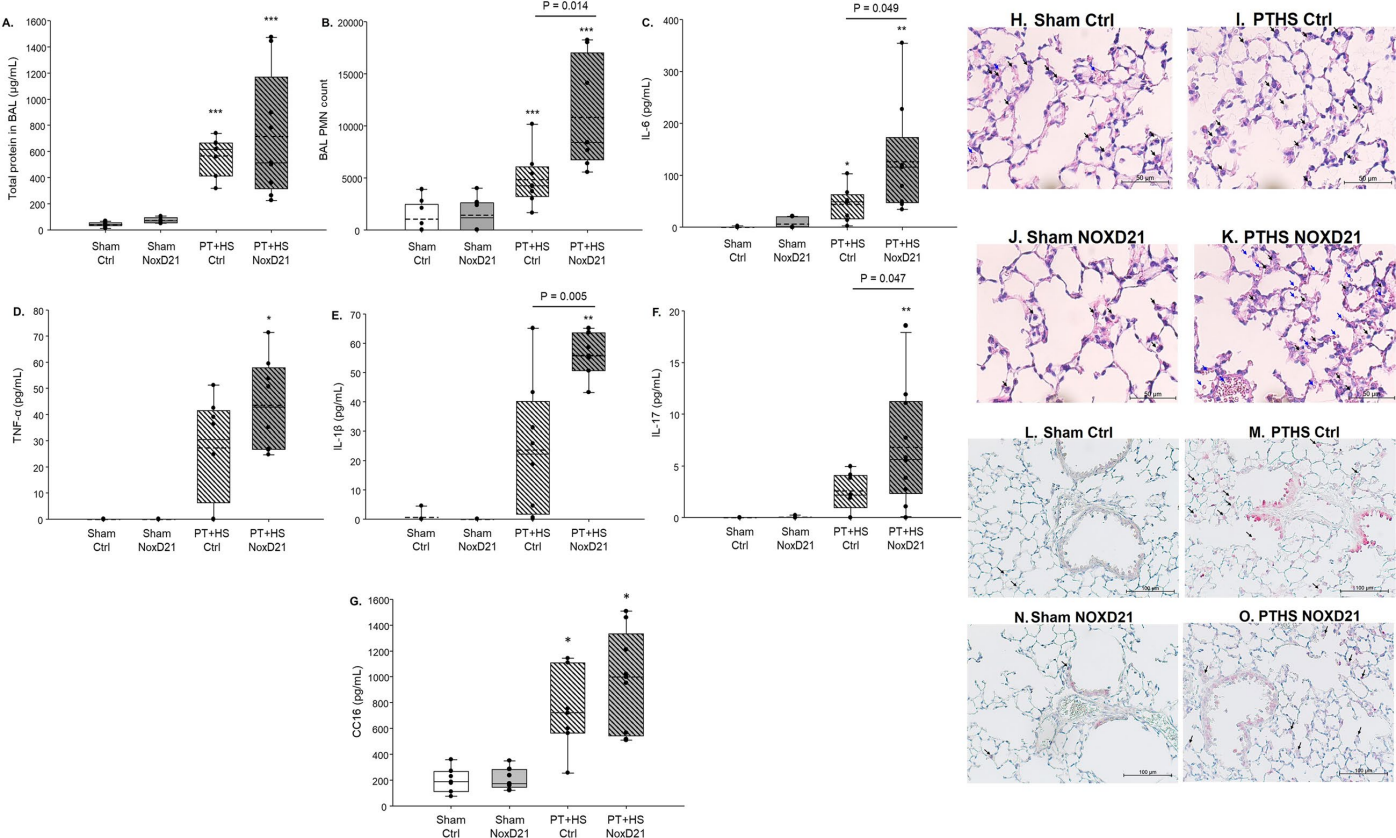
Pro-inflammatory markers and neutrophil recruitment in BAL-fluids (BALF) in PT + HS model. (**A**) Total protein in BALF. Kruskal–Wallis test with Dunn’s post-hoc analysis was performed. *** denotes p < 0.001 when PT + HS group is compared with its corresponding sham cohort. (**B**) PMN recruitment in BAL. Student’s t-test was performed to compare within PT + HS groups. PT + HS and sham animals were compared with Kruskal–Wallis test and *** denotes p < 0.001. (**C**) IL-6 content in BALF. Mann–Whitney rank sum test was performed to compare within the PT + HS groups. (**D**) TNF-α, (**E**) IL-1β and (**F**) IL-17 in BALF. For (**E**) and (**F**), Kruskal–Wallis test with Dunn’s post-hoc analysis was performed with * and ** denoting p < 0.05 and p < 0.01, respectively, when all PT + HS groups are compared to sham groups. For comparison between Ctrl-treated and NoxD21-treated PT + HS samples, Student’s t-test was performed. Animals per group for all measurements were n = 6–9. (**G**) CC16 in plasma. Two-way ANOVA with Student-Newman-Keul’s posthoc analysis was performed where * denotes p < 0.05 when PT + HS groups are compared to corresponding sham groups. Animals per group were n = 7–9. (**H**–**K**) Formalin-fixed, paraffin-embedded lung sections of Sham (Ctrl treated), Sham (NoxD21 treated), PT + HS (Ctrl treated) and PT + HS (NoxD21 treated) mice stained with H&E to characterize lung morphology. Intra-alveolar cell infiltration is indicated with a black arrow and RBCs are indicated with a blue arrow. A total of n = 8–12 lungs were stained for each group, and one representative image per group is presented. Images are at × 40 magnification. Bar = 50 µm. (**L**–**O**) Formalin-fixed, paraffin-embedded lung sections of Sham (Ctrl treated), Sham (NoxD21 treated), PT + HS (Ctrl treated) and PT + HS (NoxD21 treated) mice stained for myeloperoxidase (MPO) to detect neutrophil recruitment and activity within the lungs. Infiltrated granulocytes (marked with black arrow), alveolar epithelial cells and terminal bronchiolar epithelial cells are positively stained for MPO. A total of n = 4 lungs was stained for each group, and one representative image per group is presented. Images are at 40 × magnification. Bar = 100 µm.

As outlined, the lung is considered as a primary target and effector organ of multiple injury, particularly when a part of the injury involves blunt chest trauma. BALF was obtained to determine a range of inflammatory markers. Significantly higher levels of total protein were found in the BAL of PT + HS mice compared to sham mice, showing that C5a blockade did not rescue barrier dysfunction early after PT + HS (Fig. 2A). In addition to higher PMN in BALF in the C5a L-aptamer-treated PT + HS group (Fig. 2B), interleukin (IL)-6 (Fig. 2C), IL-1β (Fig. 2E) and IL-17 (Fig. 2F) levels were significantly higher in the NoxD21-treated compared to the Ctrl-treated PT + HS mice cohort (Fig. 2C). Despite a higher trend noted for BALF tumor necrosis factor TNF-α levels in NoxD21-treated PT + HS mice compared to Ctrl-treated PT + HS mice, this was not statistically significant (Fig. 2D). While damage of the lung was detected through circulatory Clara cell secretory protein 16 (CC16) levels, these levels were not altered due to C5a blockade within PT + HS animals (Fig. 2G).

To further estimate the impact of PT + HS on the lung, formalin-fixed paraffin-embedded lung sections were stained with hematoxylin and eosin (H&E; Fig. 2H–K)) and analyzed for neutrophil activity through MPO staining (Fig. 2L–O), Compared to the sham mice, the PT + HS mice lungs displayed thicker alveolar septa and greater immune cell infiltration, fibrin deposition and alveolar hemorrhage (Fig. 2H–K). However, there were no visual differences between Ctrl-treated and NoxD21-treated PT + HS mice lungs on histological evaluation of H&E-stained sections. Moreover, the PT + HS animal lung had both, greater immune cell infiltration (indicated with black arrows) and intra-alveolar hemorrhage, as indicated by red blood cells (RBCs) (shown with blue arrows) (Fig. 2J,K). To determine whether the recruitment and/or activity of neutrophils were altered because of NoxD21 treatment, lung sections from sham and PT + HS mice were stained for MPO (Fig. 2L–O). While sham mice stained faintly for MPO, Ctrl-treated PT + HS mice displayed higher MPO levels, primarily observed in the infiltrating immune cells, alveolar epithelial cells and the epithelial cells lining the terminal bronchioles (Fig. 2N,O). NoxD21-treated PT + HS mice lungs displayed reduced MPO staining of all the three cell types (Fig. 2O). Therefore, despite higher cytokine levels within the lung, MPO generation by neutrophils in PT + HS animals appears to be modulated due to NoxD21 treatment.

Concluding from these experiments, inhibition of C5a activity using NoxD21 appeared to elicit an enhanced pro-inflammatory cytokine response in the lungs early after PT + HS, without effecting the lung barrier dysfunction.

### Establishment of an in vitro alveolar-capillary barrier model

**Figure 3:**
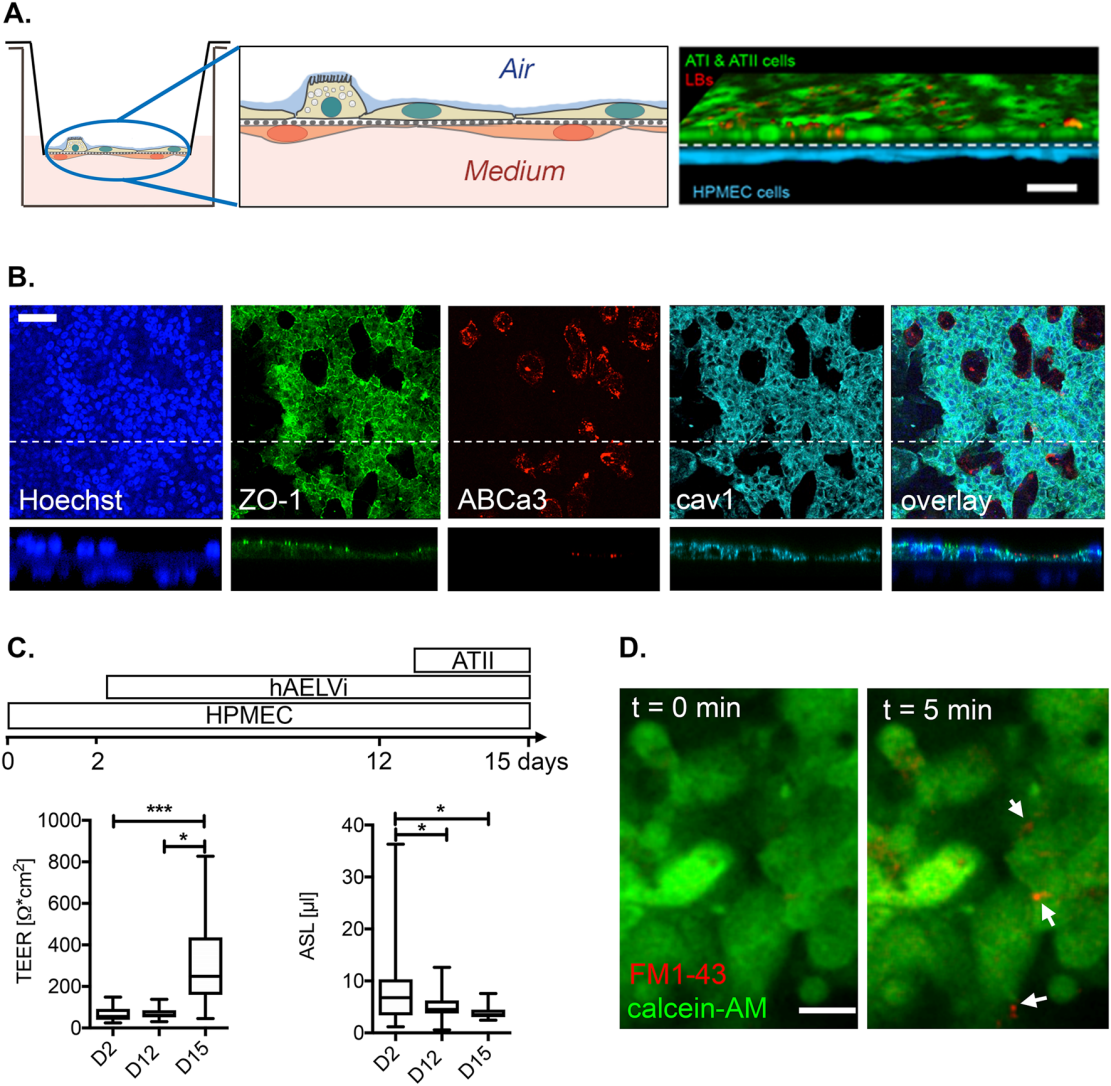
In vitro alveolar capillary barrier. (**A**) *Left, middle:* Representative scheme of the barrier model. Endothelial and alveolar epithelial cells were grown on opposing sides of permeable filter inserts. *Right:* Confocal reconstruction of endothelial (blue) and epithelial (cells) grown on filter inserts for 15 days. Staining of lamellar bodies (red) was performed with LysotrackerRed. Scale bar = 20 μm. (**B**) Immunostaining revealed expression of tight junction marker ZO-1 in epithelial cells. ABCa3 transporter was used to mark the LB membrane in ATII cells, while caveolin1 (cav1) is a marker for ATI cells and Hoechst staining indicates the nucleus. Bottom images depict a z-section through the barrier culture at the position of the dotted line in the upper images. Scale bar = 50 μm. (**C**) *Top:* Timeline of sequential seeding protocol. *Lower left:* TEER values at indicated times of culture. *Lower right:* ASL values at indicated times of culture. Values are from 18–80 individual cultures. * Indicates statistical significance when compared to day 2. (**D**) Stimulation of epithelial cells with 100 μM ATP induces exocytosis of lamellar bodies in ATII cells, indicated by the selective staining of newly secreted pulmonary surfactant with FM1-43 (arrows). Scale bar = 20 μm.

Based on the in vivo observations, we established an in vitro model of the alveolar-capillary barrier to recapitulate the cellular composition of the distal lung environment for in-depth analysis of barrier function and to specifically investigate the effects that are elicited by C5a and its effective blockade by the C5a L-aptamer. Endothelial (HPMEC-ST1.6R) and alveolar epithelial type I (hAELVi^32^) cells and primary type II (ATII)) cells were grown on opposing sides of Transwell filter inserts. A sequential seeding protocol resulted in the formation of confluent endothelial and epithelial sheets (Fig. 3A). The fully established in vitro alveolar-capillary model resembled functional characteristics of the in vivo barrier. On day 15, epithelial cells formed a tight, resorptive epithelium. The epithelial sheet composed of alveolar type I-like hAELVi cells and primary ATII cells displayed prominent expression of tight junction protein zonula occludens-1 (ZO-1), ATII marker ABCa3 and ATI marker caveolin-1 (Fig. 3B). Triple co-cultures of endothelial and epithelial cells also resulted in a significant increase in transepithelial resistance (TEER) and a significant reduction of the alveolar surface liquid (ASL) volume when compared to endothelial and endothelial-hAELVi cultures alone, respectively (Fig. 3C). The stimulation of a fully established barrier model with 100 μM ATP induced lamellar body (LB) exocytosis^33,34^, which is a functional hallmark of ATII cells^35^ (Fig. 3D).

### PMNs aggravate barrier damage in the in vitro alveolar-capillary barrier model

**Figure 4:**
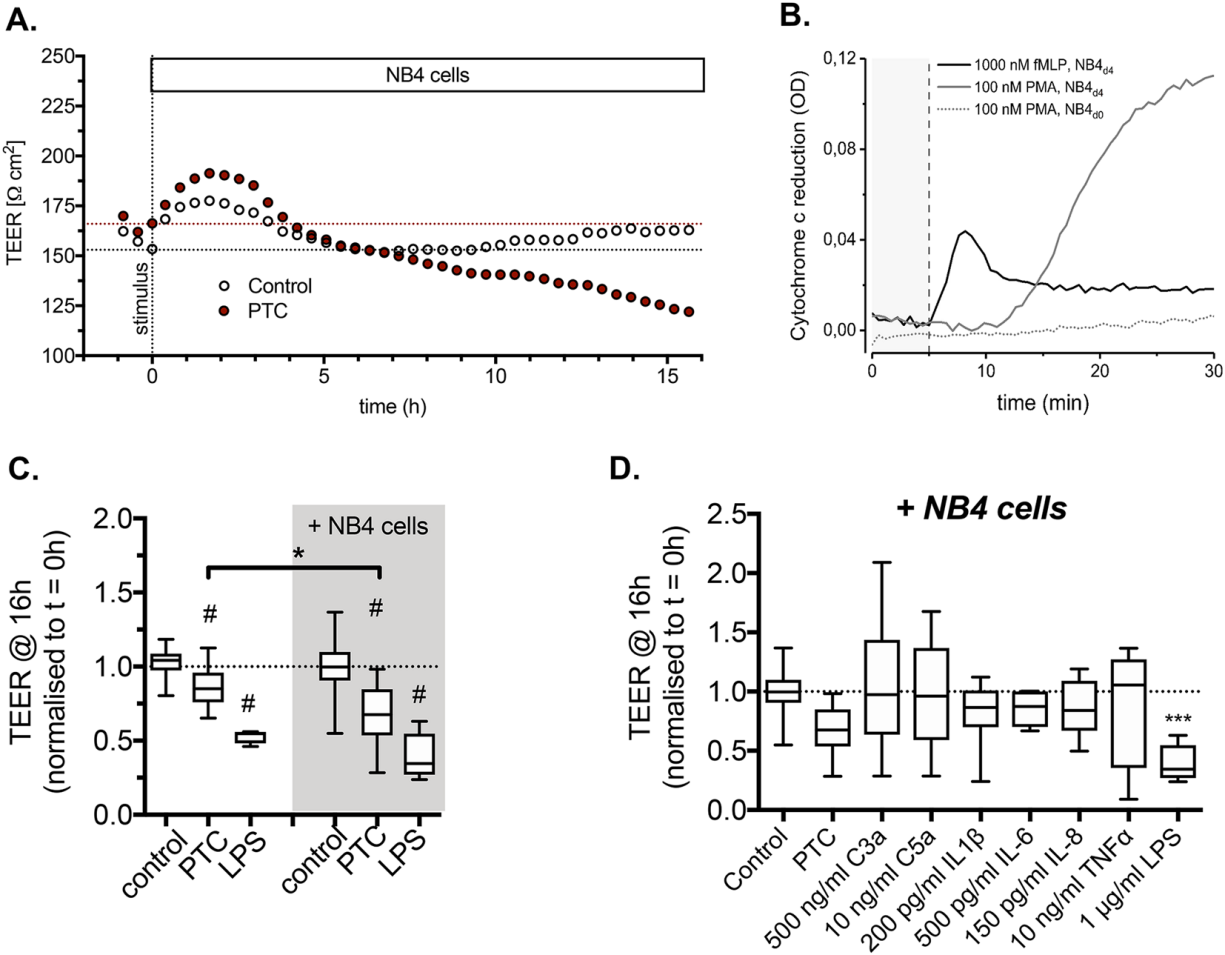
Characterization of barrier damage. (**A**) Representative TEER measurement of individual cultures in the presence of NB4 cells. The PTC (C5a, C3a, IL1β, L-6 and IL-8) was added at t = 0 (stimulus). (**B**) Oxidative burst response of neutrophil-like differentiated NB4 cells. NB4 cells were differentiated into the granulocyte lineage and primed for 30 min with 5 µM cytochalasin B before application in a SOD-inhibiting cytochrome c reduction assay. O_2_^−^ reduces Fe^3+^ of cytochrome c, which generates a color shift (increased absorbance at 545 nm compared to 535 nm). SOD consumes O_2_^−^ to produce H_2_O_2_, thereby avoiding the reduction of cytochrome c. Graph depicts difference of absorbance change in test samples versus SOD-treated control samples. PMA induces an increased O_2_^−^ production in NB4 cells differentiated for 4 days (gray solid line), while non-differentiated cells are unable to produce O_2_^−^ upon stimulation (gray dotted line). fMLP also activates a robust oxidative burst response (black line). (**C**) Quantitative analysis of changes in TEER following addition of the PTC or 1 μg/ml LPS in the absence (left) or presence (right) of NB4 cells. Data are expressed as the TEER at 16 h after stimulation normalized to the TEER at t = 0. Values are from 6–14 individual cultures. # Indicates statistical significance when compared to respective control conditions. * Indicates statistical significance when comparing cultures in the absence (left) or presence (right) of NB4 cells. (**D**) Quantitative analysis of changes in TEER following addition of C3a, C5a or individual components of the PTC in the presence of NB4 cells. Data are expressed as the TEER at 16 h after stimulation normalized to that at t = 0. Values are from 7–13 individual cultures. * Indicates statistical significance when compared to control.

To evaluate alveolar-capillary barrier damage in vitro, we monitored changes in TEER continuously to characterize barrier damage following the addition of various pathogen and damage associated molecular markers (PAMPs and DAMPs) and/or PMNs (Fig. 4A). To this end, we differentiated NB_4_ cells for 4 days, after which these cells were fully responsive to N-formyl-Met-Leu-Phe (fMLP) or phorbol 12-myristate 13-acetate (PMA) (Fig. 4B). Initial experiments revealed that the addition of a well-established C5a-containing polytrauma cocktail (PTC^36^) or of 1 μg/mL LPS resulted in an increase in TEER within the first 4 h, followed by a reduction in TEER. A robust effect of PTC or LPS addition was observed only after 16 h, when TEER was 102.7 ± 2.8% (n = 14) of the initial TEER in untreated (control) conditions and was reduced to 85.9 ± 3.8% (n = 12) and 52.8 ± 1.7% (n = 6) in cultures treated with PTC or LPS, respectively (Fig. 4C). The slower time-course compared to the in vivo model may be due to the difference in the trauma challenge (PTC instead of mechanical trauma and HS) and the difference in immune cell composition (e.g. the absence of macrophages). In response to these differences, we added primed neutrophil-like NB4 cells to the system. The presence of primed NB4 cells significantly exacerbated barrier damage in PTC-treated cultures (85.9 ± 3.8%, n = 12 vs 68.5 ± 6.5%, n = 11 in the absence or presence of NB4 cells, respectively). We also analyzed the impact of individual components of the PTC on barrier damage (Fig. 4D). However, even in the presence of NB4 cells, none of the individual factors, including C5a, resulted in a significant reduction of TEER and hence barrier damage (Fig. 4D).

### C5a-blockade does not rescue PMN- or PTC-induced damage of the alveolar barrier

**Figure 5:**
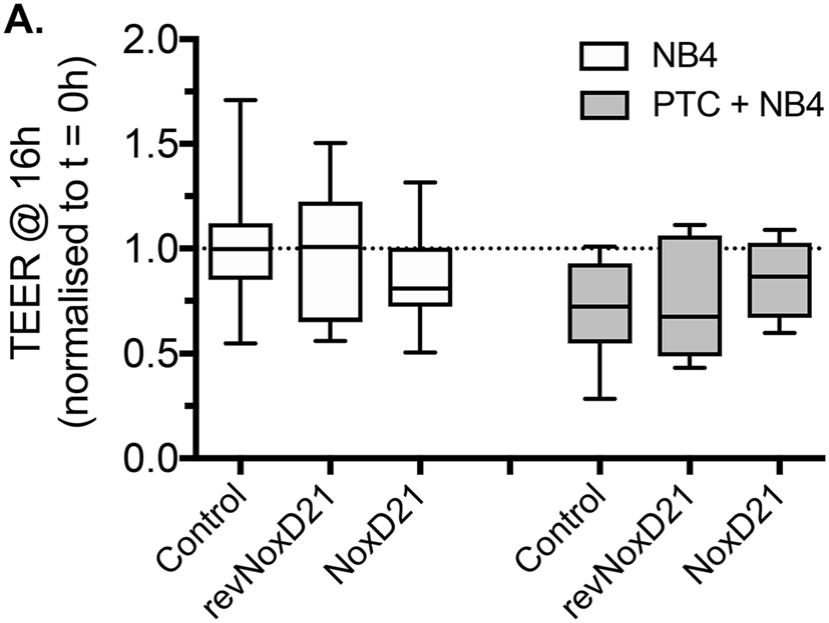
C5a L-aptamer does not rescue PMN- and PTC-induced damage of the alveolar barrier. Quantitative analysis of changes in TEER following addition of Ctrl or NoxD21 to individual cultures in the presence of NB4 cells and in the absence (left) or presence (right) of the PTC. Data are expressed as the TEER at 16 h after stimulation normalized to that at t = 0. Values are from 6–13 individual cultures.

Finally, we evaluated whether specific C5a neutralization rescues the barrier damage induced by the addition of PTC and NB4 cells. In the initial experiments, we confirmed that the C5a-neutralizing NoxD21 and Ctrl alone did not significantly affect barrier integrity. After 16 h, TEER was 85.6 ± 10.7% (n = 6) and 98.19 ± 13.9% (n = 6) of the initial TEER in cultures treated with either NoxD21 or Ctrl, respectively. When alveolar-capillary cultures were exposed to PTC and NB4 cells in the presence of Ctrl or NoxD21, TEER was not significantly increased (rescued), compared to cultures treated with PTC and NB4 alone. TEER values remained at 74.1 ± 11.92% (n = 6) and 83.3 ± 7.2% (n = 7) in the presence of Ctrl or NoxD21, respectively, compared to cultures treated with PTC and NB4 (Fig. 5). Taken together, it was shown that in the presence of NB4 cells, neither the incubation with C5a alone induces barrier reduction after 16 h nor the neutralization of C5a in PTC protects against PTC-induced barrier reduction.

### C5aR1 or C5aR2 deficiency partially resolve the inflammatory responses in the lungs early after PT + HS

While incubation with PTC for 4 h induced an initial decrease in TEER, which was further aggravated by the addition of NB4 cells, no improvement was observed when C5a was inhibited with NoxD21. Therefore, we wanted to determine what effect the absence of either C5a receptor could have in driving downstream damage and inflammatory responses after trauma injury. NoxD21 treatment can pharmacologically block the activity of C5aR1 and C5aR2 simultaneously. However, because mice which are deficient in both these receptors were not accessible to us, two different knockout variants were used, which were either deficient in C5aR1 (hereby termed C5aR1 KO) or in C5aR2 (termed C5aR2 KO).

**Figure 6:**
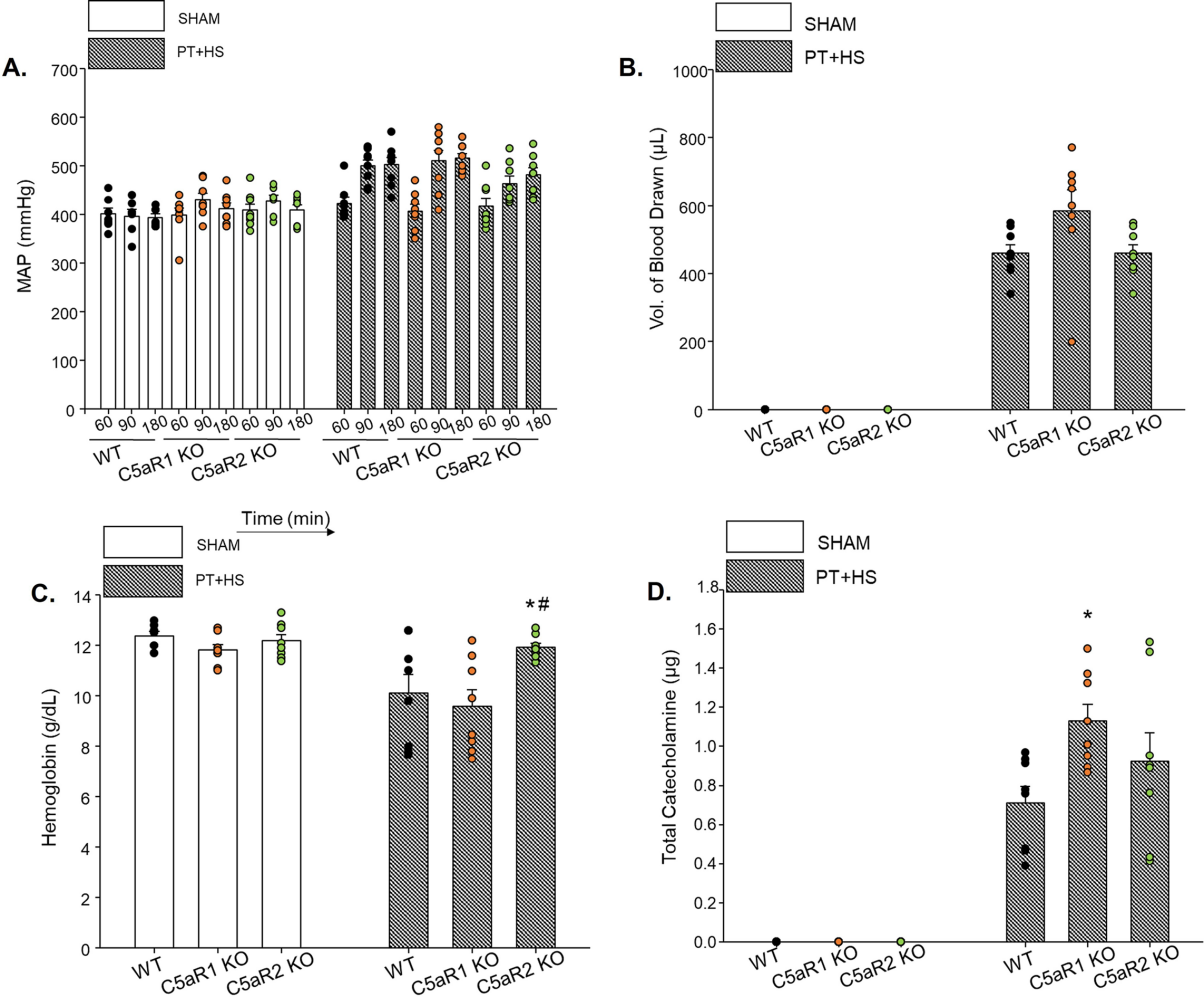
Vital status in wildtype, C5aR1 and C5aR2 knockout mice after sham or PT + HS. (**A**) MAP measurement at different time-points, (**B**) volume of blood drawn to induce HS in PT + HS animals, (**C**) blood hemoglobin and (**D**) total amount of catecholamine required to maintain blood pressure at 50 ± 5 mmHg. For (**C**), two-way ANOVA with Student-Newman-Keul’s post-hoc analysis was performed, with * and # denoting p < 0.05 for WT vs C5aR2 and C5aR1 KO vs C5aR2 KO PT + HS animals, respectively. (**D**) Catecholamine requirement to restore MAP ≥ 50 mmHg. Within the PT + HS groups, one-way ANOVA with Student-Newman-Keul’s post-hoc analysis was performed to compare between WT, C5aR1 KO and C5aR2 KO animals and * denotes p < 0.05.

**Figure 7:**
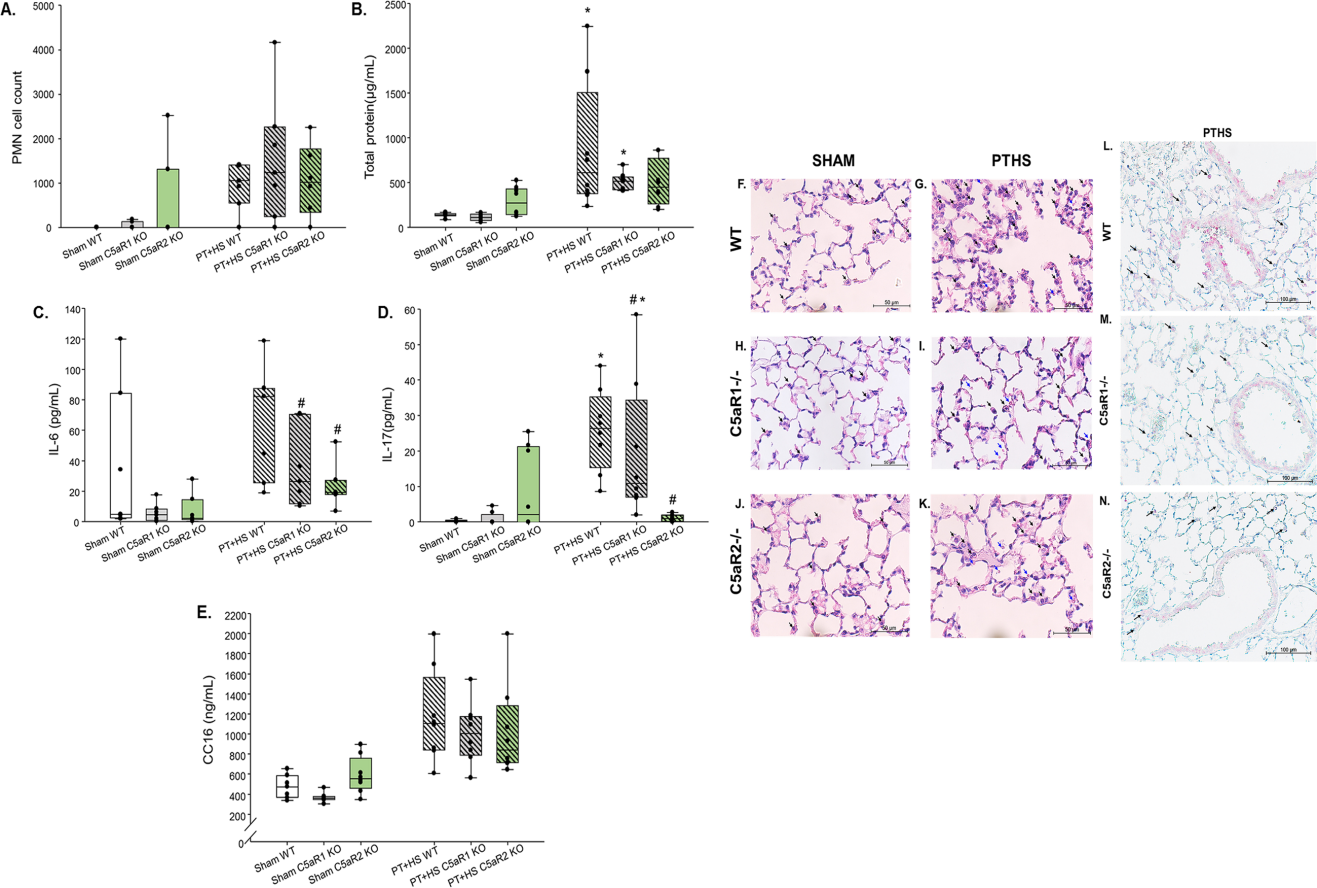
Both, C5aR1 and C5aR2 knockout PT + HS mice show attenuated BAL IL-6 and IL-17 but do not alter PMN recruitment. BAL samples were tested for (**A**) number of PMNs, (**B**) total protein content, (**C**) IL-6 and (**D**) IL-17. (**E**) Plasma CC16 levels. Kruskal–Wallis test was performed with Dunn’s post-hoc analysis, where *, denotes p < 0.05 and *** denotes p < 0.001, to compare the sham group of each genotype with the corresponding PT + HS group and # denotes p < 0.05 when compared with the WT PT + HS mice group. (**F**–**K**) Formalin-fixed, paraffin-embedded lung sections of Sham (WT), Sham (C5aR1 KO), Sham (C5aR2 KO), PT + HS (WT), PT + HS (C5aR1 KO) and PT + HS (C5aR2 KO) mice were stained with H&E staining to characterize lung morphology. Intra-alveolar cell infiltration is indicated with a black arrow and RBCs are indicated with a blue arrow. A total of n = 8–12 lungs were stained for each group, and one representative image per group is presented. Images are at 40 × magnification. Bar = 50 µm. (**L**–**N**) Formalin-fixed, paraffin-embedded lung sections of PT + HS (WT), PT + HS (C5aR1 KO) and PT + HS (C5aR2 KO) mice stained for MPO to detect neutrophil recruitment and activity within the lungs. Infiltrated granulocytes (marked with black arrow), alveolar epithelial cells and terminal bronchiolar epithelial cells are positively stained for MPO. A total of n = 4 lungs were stained for each group, and one representative image per group is presented. Images are at × 40 magnification. Bar = 100 µm.

Age- and sex-matched male C57bl6 wildtype (WT), C5aR1 KO and C5aR2 KO mice underwent PT + HS or a sham operation. In the early phase after multiple traumatic impact, the MAP and volume of blood drawn to induce HS were not different within WT or C5aR-deficient mice of the PT + HS groups (Fig. 6A,B). The total demand of catecholamines of the C5aR-deficient PT + HS mice was comparatively higher than the WT PT + HS mice (Fig. 6D). Because approximately 20% less blood was drawn from the C5aR2 KO PT + HS mice than from the C5aR1 KO PT + HS group, the amount of hemoglobin was almost 20% higher within the C5aR2 KO PT + HS animals (Fig. 6C). The total protein concentration (Fig. 7A) and PMN count (Fig. 7B) in BALF did not vary between the various PT + HS treatment groups. IL-6 levels (Fig. 7C) were significantly attenuated in the absence of C5aR2 (Fig. 7C), and when tested for IL-17 (Fig. 7D), a significant decrease could be detected in the BALF of C5aR2 KO PT + HS animals when compared to WT PT + HS mice. As found previously with pharmacological C5a inhibition, the absence of C5aRs did not affect the circulatory levels of the lung damage marker CC16 (Fig. 7E). The lung morphology post PT + HS could again be characterized by edematous alveolar septa, more immune cells and RBC infiltration in WT, C5aR1 KO and C5aR2 KO mice (Fig. 7F–K). As observed within NoxD21-treated PT + HS mice (Fig. 2L–O), the absence of C5aR1 or C5aR2 resulted in less MPO staining in the infiltrated granulocytes and the alveolar and terminal bronchiolar epithelial cells of the lung of PT + HS mice (Fig. 7M,N) when compared to the WT mice (Fig. 7L). Because sham lungs were negative for MPO staining, WT, C5aR1 KO and C5aR2 KO sham mice lungs have not been presented.

## Discussion

Although experimental and clinical evidence indicate a rapid and robust activation of the complement system after severe injury^5,6,37^, there is no empirical evidence whether C5a inhibition is protective early after PT + HS. In this study, a PEGylated C5a L-aptamer Nox-D21 that inhibits C5a activity was used in an experimental model of PT + HS. Following confirmation that the aptamer could inhibit C5a-mediated PMN chemotaxis in vitro (Fig. 1A), a well-established standardized murine model of PT + HS was used^11^ in the presence or absence of an early posttraumatic C5a inhibition by NoxD21 (Fig. 1.–F; Fig. 2). In general, the lungs can be considered as the central hub of the complement-driven inflammatory response after trauma^2^. Early neutralization of C5a may result in reduced recruitment of immune cells into the alveolar space, as observed up to 24 h after injury in our rat blunt chest trauma model^19^. By contrast, blocking C5a alone did not reduce, rather enhanced the PMN count within the BALF of NoxD21-treated PT + HS mice 4 h after the trauma impact (Fig. 2). However, the effector function of neutrophils, measured by MPO staining, was significantly reduced after NoxD21 treatment within the PT + HS mice group (Fig. 2O). This could indicate that while C5a blockade impaired the oxidative burst of the neutrophils, it may have resulted in an indirect increment in pro-inflammatory responses because this blockade targeted both, C5aR1 and C5aR2 concomitantly. In an in vitro setting, Haegens et al., demonstrated that bronchiolar as well as alveolar epithelial cells can take up MPO from their extracellular environment, which can inhibit the synthesis of proinflammatory cytokines like IL-6 and IL-8 by these cells^38^. This report excluded the presumption that transcriptional regulation of NF-kB or AP-1 could be altered by MPO and could not offer a mechanistic insight as to why MPO decreased cytokine synthesis by lung epithelial cells. Therefore, we could not conclude unambiguously whether NoxD21-mediated inhibition of PMN-MPO generation did indeed result in increased BALF cytokines synthesis locally by the lung epithelial cells. With consequent proinflammatory responses, an early barrier damage could not be detected shortly after PT + HS, as indicated by similar levels of circulatory CC16 levels in the Ctrl and NoxD21-treated PT + HS animals (Fig. 2G). There were a few limitations to this in vivo study. The BALF samples obtained could have been contaminated by blood (because of the blunt chest trauma), which may contribute to the neutrophil content and cytokine levels measured within BALF. To circumvent this, H&E staining of formalin-fixed lung sections was applied where significant RBC and immune cell infiltration were noted within the PT + HS mice, without any difference between Ctrl or NoxD21 treatment (Fig. 2H–K). Therefore, to gain a deeper insight into lung-associated barrier damage, an in vitro three-dimensional standardized alveolar barrier model was used.

The alveolar-capillary barrier model was established to obtain a reproducible system that closely mimics the physiological properties of the alveolus (cellular composition, air–liquid interphase, epithelial and endothelial properties). This model provided the advantage of having all three cell-types—hAELvi alveolar type I (ATI) and primary ATII cells and primary human endothelial cells (HPMECST1.6R cells)—to functionally simulate the alveolar space^32,39–43^ (Fig. 3). Fragile primary ATI cells frequently become damaged during processing, whereas the hAELVi (ATI) cell line has proven to display type I-like characteristics and functional tight junctions and maintains a stable barrier at air–liquid culture conditions for extended proliferation cycles. Because HPMECs display high batch-to-batch variability, significant levels of contamination (i.e., mainly fibroblasts) and constitutive activation of inflammatory responses during culturing, HMPECSTR1.6R cells were used. In vitro treatment with the “polytrauma cocktail”^26^ and not with each singular component, particularly in the presence of NB4 cells, led to reduced TEER values only after 16 h (Fig. 4). This could partially explain why an exclusive “early” barrier damage within NoxD21-treated PT + HS animals was not detected (Fig. 2G.), which may be exacerbated in the same experimental model when observed at a later time-point. What advantage the singular blockade of C5a has on PT + HS-associated SIRS requires further investigation. The role of recruited monocytes was also not considered within the in vitro experiments, which impairs a holistic understanding of what occured locally within the lungs in our in vivo PT + HS model.

Because HS has been proven to drive the severity of the SIRS response^11,12^, NoxD21 was administered prior to HS induction in the PT + HS animals (Fig. 1B). To determine whether deficiency of the two C5a receptors have distinct effects, two different knock-out mice were used (Figs. 6 and 7). The singular and combined effects of C5aR1 and C5aR2 have been elucidated in a range of biological responses^44–46^. Mice deficient in C5aR1 or C5aR2 were used in this regard to distinguish between their effects, because NoxD21 practically blocks the activity of both these receptors concomitantly. The absence of C5aR2 not only reduced MPO secretion by PMNs (Fig. 7L–N), but also alleviated IL-6 as well as IL-17 levels within the PT + HS animals (Fig. 7A,B). Therefore, contrary to the absence of both the receptor activities (Fig. 2C,D), overcoming the effect of C5aR2 alone may prove to be beneficial in resolving local lung inflammation post trauma injury. What effect this may confer in long-term barrier dysfunction responses, should be addressed accordingly in the future.

This study indeed provokes further questions. Firstly, the blockade of upstream complement factors and its activation products (like C3 and anaphylatoxin C3a) could further help resolve how they affect PT + HS-associated SIRS responses. Secondly, the subsequent SIRS and the respective blockade of complement activation products may affect each organ differently and may depend on the type of injury. When C5aR2-deficient mice were first engineered, the authors also described the relevance of C5aR2 activity abrogation helping in the resolution of local inflammation in an experimental model of pulmonary immune complex injury^47^. Subsequently, Kovtun et al. showed that an experimental model of combined thorax trauma and bone fracture injury resulted in enhanced BALF TNF-α, IL-10 and CXCL-1 levels in C5aR2 KO mice^48^. The absence of C5aR1 or C5aR2 activity did not alter BALF IL-6 levels^48^. As implied by the results of this report, the inflammatory response in the lung was driven primarily by thorax trauma, and necessary comparisons cannot be drawn with our study because of differences in the cytokines monitored as well as the type of injury, which in our model includes the additional impact of HS. Thirdly, a robust comparison between the early versus delayed effects of complement blockade after PT + HS should be undertaken in the future. This will help address questions in the context of how barrier dysfunction develops due to inflammatory responses and whether this contributes to complications like sepsis and MODs in the later stages of trauma injury. Fourthly, a combination of these approaches may help to understand the basis of the striking differences in C5a inhibitory strategies in different SIRS models. This offers the opportunity to determine under what circumstances complement blockade is detrimental and/or recuperative^49^. Presumably, such findings may help explain direct or even indirect alterations of the pulmonary inflammatory profile following polytrauma and the damage which consequently occurs, helping to initiate further research efforts in elucidating these yet unknown mechanisms.

## Materials and methods

### Neutrophil isolation

For in vitro PMN experiments, approval was received from the Independent Local Ethics Committee of the University of Ulm (Ethic approval number 69/80). Blood from adult healthy volunteers was obtained through informed and written consent. For the procurement of blood and experiments thereof, all regulations were adhered to as stipulated by the local ethics committee. Blood was obtained in tubes containing 10% citrate dextrose (Sarstedt, Germany), the blood was layered on Ficoll-Paque (Amersham Biosciences) and subjected to gradient centrifugation, followed by dextran sedimentation and hypotonic lysis of residual erythrocytes. The desired cell suspension of neutrophils was prepared in Dulbecco’s phosphate-buffered saline (DPBS, Life Technologies, USA).

### Chemotaxis assay

Firstly, a PMN cell suspension was prepared by dissolving a maximum of 10 × 10^6^ cells per 2 mL DPBS + 0.1% bovine serum albumin (BSA). A stock of BCECF (2′,7′-bis [2-carboxyethyl]-5-[and 6]-carboxy-fluorescein acetoxymethyl ester; Molecular Probes, USA) was dissolved first in 50 µL dimethyl sulfoxide (DMSO). Following dissolution, 1.6 µL diluted BCECF was added to each mL of cell suspension. Cells were then incubated in a water-bath, at 37 °C, for 30 min at 50 rpm. Following incubation, the cell suspension was centrifuged at 340×*g*, at room temperature (RT), for 5 min. The supernatant was discarded, and the cells were diluted in Hank’s Balanced Salt Solution (HBSS) at 5 × 10^6^ cells/mL. At the lower chamber of the Boyden Chemotaxis instrument, 33 µL of the following solutions were loaded: HBSS alone, C5a alone (100 ng/mL; corresponding to 12 nmol/L), revNoxD21 or NoxD21 alone (100 µg/mL; corresponding to 7.6 µmol/L), C5a (100 ng/mL) + revNoxD21/NoxD21 (10 µg/mL; corresponding to 0.76 nmol/L) and C5a (100 ng/mL) + revNoxD21/NoxD21 (100 µg/mL). For preincubated conditions, revNoxD21 or NoxD21 at 10 µg/mL or 100 µg/mL concentrations were incubated with C5a (100 ng/mL) and then loaded into the wells of the lower chamber. Following secure placement of the chemotaxis membrane (Polycarbonate Filters (3 µm); Neuro Probe), the upper-chamber was positioned above, where 44 µL BCECF-incorporated PMN cell suspension was added. After incubating this setup for 30 min at 37 °C, the membrane was washed with DPBS ++ (Gibco, USA) + 0.1% BSA and left to dry in the dark. Finally, the membrane was measured using Fluoroskan Ascent (Thermo Fisher Scientific, USA) at 485/538 nm.

### Murine poyltrauma and hemorrhagic shock model

The animal studies were performed after the review and approval of the regional authority (Regierungspräsidium Tübingen, Germany, registration number 1194). The model has been previously described in the study of Denk et al.^11^. All procedures were performed as approved by the Regierungspräsidium and all regulations were followed. Male 8–12-week-old C57Bl6 (WT), C5aR1 KO (C5aR1 KO) and C5aR2 KO mice with a mean weight of 28–33 g were used with n = 8–12 per group. The studies were performed in two phases; first to test the efficacy of NoxD21 and then to determine the effect of C5aR1 or C5aR2 deficiency.

C5a L-aptamer (NoxD21) or a control (Ctrl; rev NoxD21) was used in sham operated (Sham) or polytrauma with hemorrhagic shock (PT + HS) mice. As per our model, each mouse was anesthetized using 3% sevoflurane (Sevorane Abbott, Germany)/97.5% oxygen. For analgesia, buprenorphine was injected subcutaneously at 0.05 mg/kg bodyweight prior to PT induction. PT, as a combined injury of thorax trauma, traumatic brain injury and femoral fracture with soft tissue injury was performed over a time-span of 20 min. We consider the time-point at which the PT was performed as 0. Following this, a venous catheter was inserted into the right jugular vein, through which 200 µL (10 mg/kg bodyweight) of NoxD21 or Ctrl was injected. Thereafter, an arterial catheter was inserted in the left femoral artery, and heparin (Heparin-Natrium; B. Braun, Germany) was injected through this catheter at 2 IU/mouse. Ischemia was induced 60 min after PT by drawing blood until the MAP fell to 30 ± 5 mmHg. After 1 h, a bolus of 400 μL Jonosteril (Fresenius Kabi, Germany) was given and the mouse was resuscitated with four times the volume of blood drawn during ischemia to restore the MAP to 50 ± 5 mmHg over 30 min. When the MAP fluctuated below 50 mmHg, norepinephrine was provided (0.5–2 μg/kg/min) to maintain the aforementioned MAP. Sham mice did not receive PT or HS but were catheterized with arterial and venous lines and received the bolus as described above. The vital status, including heart rate, MAP, catecholamine requirement, temperature and sevoflurane flow, was recorded every 5 min throughout the entire duration of the study after instrumentation. At 4 h post-PT + HS, the mice were euthanized by sevoflurane overdose followed by cardiac puncture.

For C5aR1 KO and C5aR2 KO mice, the same procedures were performed with groups assigned as follows; Sham (WT), Sham (C5aR1 KO), Sham (C5aR2 KO), PT + HS (WT), PT + HS (C5aR1 KO) and PT + HS (C5aR2 KO). Each group contained n = 8–12 animals and PT + HS or sham was performed as aforementioned.

### Bronchoalveolar lavage (BAL)

Following sacrifice, a ligation on the left lung was provided and the right lung was washed three times with 500 μL DPBS. The first wash was collected in a tube containing protease inhibitor (Sigma Aldrich, USA) and the final two washes were combined and collected in a second such tube. The BALF was centrifuged at 400×*g* for 10 min at 4 °C. Supernatant was collected from the first tube and used for measuring total protein and proinflammatory cytokines. Supernatant from the second tube was discarded. The cell pellets from the two tubes were pooled in a total volume of 100 μL DPBS and 50 μL from this suspension was used to prepare a cytospin. Cells were counted in a Neubauer Cell Counting chamber by staining the cells with crystal violet. Cytospins were stained using Hemacolour Rapid Staining kit (EMD Millipore, USA) as per the manufacturer’s protocol and then neutrophils were counted.

### Total protein estimation

Collected supernatants from BAL were tested for total protein using the BCA Protein Assay Kit (Thermo Fisher Scientific, USA) as per the manufacturer’s protocol.

### Enzyme linked immunosorbent assay (ELISA)

ELISAs were performed with pre-coated kits and as per the manufacturers’ directions to test for proinflammatory cytokine markers for mouse IL-6 (BD Biosciences, USA), TNF-α (BD Biosciences, USA), IL-1β (BD Biosciences, USA), monocyte-chemoattractant protein-1 (MCP-1) (BD Biosciences, USA), IL-17 (R&D Systems, USA) and keratinocytes-derived chemokine (KC) (R&D Systems, USA).

### Immunohistochemistry

Lungs from sham and PT + HS mice were formalin fixed for a minimum of 24 h and embedded in paraffin after dehydration with ethanol and xylol. Paraffin-embedded lung sections of 4 µm were prepared of all animals in each experimental group on glass slides, stained with H&E and visualized using a Zeiss Axio Imager A1 microscope (Zeiss, Germany).

### MPO staining

To test for MPO, 4 µm lung sections were cut and rehydrated and the antigen was retrieved by boiling in citrate buffer (pH 6.5) for a total of 7 min ensuring that the slides are completely immersed in the buffer throughout. The sections were incubated overnight at 4 °C with an MPO primary antibody (Abcam, UK) at 1:25 dilution. Following washing, goat anti-rabbit horse-radish peroxidase (HRP)-labeled secondary antibody (Abcam, UK) was added at 1:50 dilution at RT for 30 min and developed with DAKO (Agilent, USA) for 20 min, dehydrated and mounted with a glass coverslip. The sections were visualized using a Zeiss Axio Imager A1 microscope (Zeiss, Germany). For all experimental groups, n = 4 was used.

### Cell culture

HPMEC-ST1.6R (HPMEC; gifted by Prof. Kirkpatrick, Mainz, Germany) cells were maintained in M199 medium (Sigma-Aldrich, Germany) supplemented with 10% charcoal-stripped fetal bovine serum (FBS, GE Healthcare, USA), 1% GlutaMAX (Thermo Fisher Scientific, USA), 1% PenStrep (Thermo Fisher Scientific, USA), 25 μg/mL endothelial cell growth supplement and 25 μg/mL heparin in cell culture flasks coated with 0.2% gelatin. Cells were used for experiments up to a maximum of 35 passages. hAELVi cells (InSCREENeX, Germany)^32^ were maintained in huAEC medium (InSCREENeX, Germany) supplemented with 1% PenStrep in cell culture flasks coated with huAEC coating solution (InSCREENeX, Germany). ATII cells were isolated from lung tissue according to the procedure of Dobbs et al.^50^ with minor modifications as recently described^51^. NB4 cells were maintained in RPMI 1640 containing 25 mM HEPES (Irvine Scientific, USA), 10% FBS and 1% PenStrep and subcultured to a maximum of 20 passages. NB4 Cells were differentiated into the granulocyte cell type by treatment with 10 μM all-trans-retinoic acid (Sigma-Aldrich, Germany) for 5–7 days before use in alveolar capillary barrier models^52^ and potentiated with N,N’-hexamethylene bis(acetamide) (Sigma-Aldrich, Germany) on day 1 after initiation^53^.

Alveolar capillary barrier models were established on Transwell filter inserts (Corning Costar 3470; Corning Inc., USA). In brief, filters were precoated with 50 μL of a 30 μg/mL collagen solution and left to dry overnight at room temperature. The collagen was subsequently cross-linked in an ultraviolet oven for 30 min. 5 × 10^4^ HPMEC cells were seeded onto the basolateral side of inverted filters, allowing them to attach for 2 h, after which the filters were turned into an upright position and maintained in M199 medium for 48 h. The apical side of the filters were then coated with huAEC-coating solution, and 7.5 × 10^4^ hAELVi cells were seeded on this side in huAEC-Medium. After 24 h, the filters were set to air–liquid interface (ALI) conditions by removing the apical fluid and maintained in huAEC medium for 10 days. Freshly isolated ATII cells were seeded on top of the the hAELVi cell layer and allowed to integrate for 24 h before cultures were again set to ALI conditions. Fully established alveolar capillary barrier models were used for experiments after 24 h.

### O_2_^−^ production by NB4 cells

The method is based on the superoxide-dismutase (SOD) inhibitable cytochrome c reduction assay as previously described^54^ with slight modifications. Briefly, 100 µM cytochrome c (Sigma-Aldrich, Germany) were mixed with either SOD (Sigma-Aldrich, final activity 250 U/mL) for control or HBSS with Ca^2+^ and Mg^2+^ (HBSS ++ ; Thermo Fisher Scientific, USA) for test samples. A total of 2 × 10^5^ cytochalasin B-primed (5 µM, 30 min, Sigma-Aldrich, Germany) NB4 cells suspended in HBSS ++ were added to each test well of a 96-well tissue-culture treated microtiter plate (Sarstedt, Germany). Optical density was determined on a plate reader (Tecan Trading AG, Switzerland) at wavelengths of 545 and 535 nm in a kinetic cycle for at least 30 min at 37 °C. After 5 min, 1 µM fMLP (Sigma-Aldrich, Germany) or 100 nM phorbol-myristate-acetate (Sigma-Aldrich, Germany) were added.

### Measurement of the transepithelial electrical resistance (TEER)

TEER was analyzed by impedance spectrometry using the cellZscope (NanoAnalytics, Germany). For measurements, a filter was inserted after basal electrodes were overlaid with 500 µL equilibrated huAEC medium and 260 µL of this medium was further added to the apical side of the filter. Measurements were performed immediately after positioning of apical electrodes. Data were acquired and analyzed using the software provided with the instrument (NanoAnalytics, Germany).

### Measurement of the apical surface liquid (ASL) volume

ASL measurements were performed using the D_2_O dilution method as described previously^55^. In brief, apical aqueous volumes were diluted by mixing with 25 µL 0.9% (w/v) NaCl and combined with 25 μL D_2_O containing 0.9% (w/v) NaCl for analysis. Water concentrations were determined by attenuated total reflection mid-infrared spectroscopy to calculate ASL volumes. This method enables the measurement of minute aqueous volumes with a resolution of < 25 nl.

### Materials for staining ATII cells and ZO-1

Mouse anti-P180 lamellar body protein (ABCa3), goat anti-ZO-1 and rabbit anti-caveolin-1 antibodies were purchased from Abcam, UK. Fluorescently labelled secondary antibodies and fluorescent dyes were obtained from Molecular Probes (Thermo Fisher Scientific, USA). All chemicals were purchased from Sigma-Aldrich, Germany, unless stated otherwise.

### Immunofluorescence

For immunofluorescence staining, cells were washed twice in PBS (pH 7.4, Biochrom, Germany), fixed for 20 min in 4% paraformaldehyde in PBS, washed thrice in PBS and permeabilized for 10 min with 0.2% saponin and 10% FBS (Thermo Fisher Scientific, USA) in PBS. Cells were subsequently stained with primary (1:100) and secondary (1:400) antibodies in PBS, 0.2% saponin, 10 mM HEPES and 10% FBS. Images were obtained using an inverted confocal microscope (Leica TCS SP5, Leica, Germany) with a 63 × lens (Leica HCX PL APO 63.0 /.3 GLYC CORR CS). Images for the blue (Hoechst), green (AlexaFluor 488), red (AlexaFluor 568) and far-red (AlexaFluor 647) channels were taken in sequential mode using appropriate excitation and emission settings.

### Live-cell fluorescence imaging of lamellar body (LB) exocytosis

FM1-43 experiments to detect individual LB fusion events^33^ were performed using an inverted confocal microscope (Leica TCS SP5, Leica) using a 63 × lens (Leica HCX PL APO 63.0/1.3 GLYC CORR CS)^56^. For combined calcein-AM and FM1-43 experiments (both from Thermo Fisher Scientific, USA), cells were loaded for 20 min with calcein-AM (1 µM) before the start of the experiment, washed three times in PBS and mounted in bath solution (140 mM NaCl, 5 mM KCl, 1 mM MgCl_2_, 2 mM CaCl_2_, 5 mM glucose, 10 mM Hepes, pH 7.4) containing 1 µM FM1-43 within an eight-well slide (IBIDI, Germany). LB exocytosis was induced by the addition of ATP (100 μM) to the apical side of the filters.

### Statistics

All statistics and graph production were performed in Microsoft Excel (Microsoft, Redmond, USA) or GraphPad Prism 5 (GraphPad, USA) for the in vitro studies, and with SigmaPlot 14.0 (Systat Software Inc., USA) for the PMN chemotaxis experiment and in vivo studies. The data is presented as the mean ± standard error of the mean, unless noted otherwise. After testing for normality, datasets were tested for outliers with Grubb’s outlier test and these data points were removed. For comparison of two independent parametric datasets, an unpaired two-tailed Student’s t-test was used and for non-parametric dataset, Mann–Whitney rank sum test was applied. For multiple parametric datasets, statistical significance was determined using two-way analysis of variance and Student-Newman-Keul’s post-hoc analysis. For non-parametric datasets, Kruskal–Wallis test followed by Dunn’s post-hoc analysis was performed as indicated. When data is represented as box and whisker plots superimposed with scatter plots: the lower margin refers to the 25th percentile; the upper margin refers to 75th percentile; the solid line within the box refers to the median; the whiskers demarcate the upper and lower limits. For all graphs, shaded bar plots and box plots refer to the PT + HS treatment groups.

## Supplementary Information

Supplementary Information.
