# Supplementary material for: Role of the C5a-C5a receptor axis in the inflammatory responses of the lungs after experimental polytrauma and hemorrhagic shock
Source: Sci Rep. 2021 Jan 25;11:2158. doi: 10.1038/s41598-020-79607-1 (PMC7835219; doi:10.1038/s41598-020-79607-1)
Supplement: Supplementary file 1 — Supplementary Information. [file 41598_2020_79607_MOESM1_ESM.docx]

**Role of the C5a-C5a receptor axis in the inflammatory responses of the lungs after experimental polytrauma and hemorrhagic shock.**

Shinjini Chakraborty, Veronika Eva Winkelmann, Sonja Braumüller, Annette Palmer, Anke Schultze, Bettina Klohs, Anita Ignatius, Axel Vater, Michael Fauler, Manfred Frick and Markus Huber-Lang

**Supplementary information**

Genotyping

DNA extraction was performed, and PCR products were prepared from tail clippings of C5aR1 KO and C5aR2 KO C57bl6 mice using the REDExtract-N-Ampa Tissue PCR Kit (XNAT-100RXN, Sigma-Aldrich, Germany) following the manufacturer’s protocol. The respective genes *C5ar1* [1] and *C5ar2* [2] on mouse chromosome 7 were targeted. For *C5ar1*, the forward primer used was - 5'GGCCATCCTGCGGCTGATGG 3' and the reverse primer used was -5'GCCTTGCGACTCCAGGTCCG3'. For *C5ar2,* five different primer pairs were tested. These primer pairs were as follows: -

Pair AI - forward primer: 5'CACACCACCAGCGAGTATTATG3' and reverse primer 5'AGCACAAGCAGGACTATCAGG3'.
Pair 1 - forward primer: 5'AGCACTATCCTCCCCGACTT3' and reverse primer 5'GATACCTTGGTCACCGCACT3'.
Pair 2- forward primer: 5'TACTGAGCTCCACCCCAAAC3' and reverse primer:
5'AAGGCCTCTTCCATCCTTGT3'.

Pair 3 - forward primer: 5'GTCTGGGGGCCAGAATGAT3' and reverse primer:
5'GCACACCCACCAGGAAGAC3'.

Pair 4 - forward primer: 5'GTGTCTGGGGGCCAGAAT3' and reverse primer:
5'CTGTGGCGGGACTCTTTC3'.

For Neomycin, the forward primer used was - 5'CCTGTCCGGTGCCCTGAATGAA3' and the reverse primer used was - 5'GCCGATCCCCTCAGAAGAAC3'.

The amplification procedure was as follows; for *C5ar1*, a denaturation step of 5 min at 96°C, then 32 cycles of 30 sec at 94°C, 30 sec at 71°C and 1 min at 72°C, and concluding with a final extension step of 7 min at 72°C. For *C5ar2* (for all primer pairs used) and neomycin, a denaturation step of 5 min at 96°C, then 32 cycles of 30 sec at 94°C, 30 sec at 60°C and 1 min at 72°C, and concluding with a final extension step of 7 min at 72°C. All PCR products were visualized on 1.5% agarose gel containing 1 µg/ml of ethidium bromide.

*C5aR1 KO mice genotyping*

The C5aR1 KO mouse was originally generated by Gerard et al. [1]. These mice were initially found to display no amplification product when tested for *C5ar2* using the primer pair AI (Figure S1). However, when four different primer pairs were tested (Pairs 1, 2, 3 and 4), the C5aR1 KO mouse tail extracts showed amplification products for all the tested pairs (Figure S2; data for primer pair 2 not shown). Moreover, when tested with primer combinations - a) forward primer from pair 1+reverse primer from AI and b) forward primer from pair AI+reverse primer from pair 3 (Figure S3), *C5ar2* amplification product was difficult to be detected within C5aR1 KO mice tail extracts for the first combination (Figure S3. A.). This was not the case for the second combination used (Figure S3. B.). Consequently, it was realized that contrary to our initial conclusion, the C5aR1-deficient mice used were specifically deficient in C5aR1 and sufficient in C5aR2 activity.


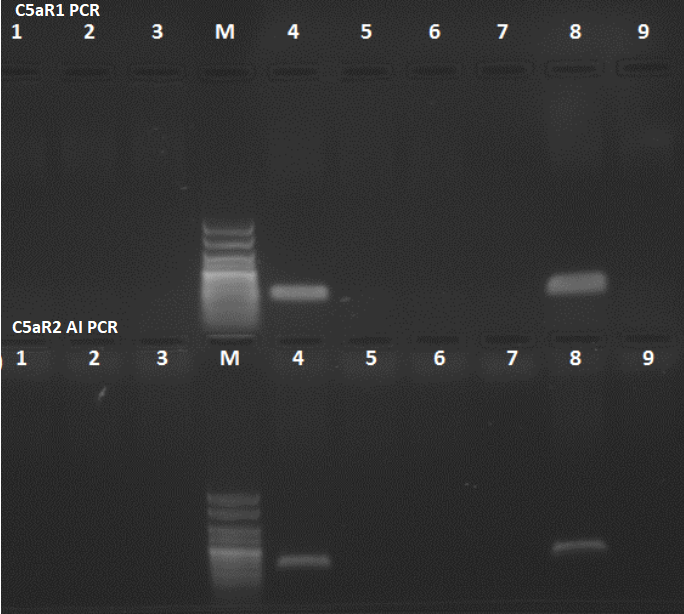


Figure S1: Genotyping C5aR1 KO mice with *C5ar1* and *C5ar2* primers. C5aR1 KO mice were tested for *C5ar1* with the aforementioned primer, and also for *C5ar2* with primer A.I. Lanes 1, 2, 3, 5, 6 and 9 were loaded with DNA extracted from C5aR1 KO mice, lane 7 corresponds to water control and lanes 4 and 8 were loaded with extracts from WT mice. M refers to the loaded markers.


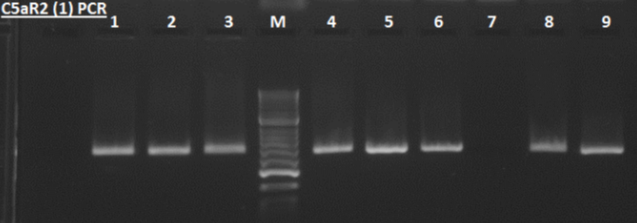

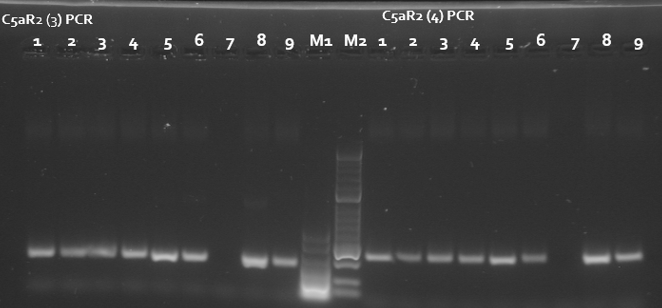


Figure S2: Genotyping C5aR1 KO mice with *C5ar2* primers. C5aR1 KO mice were tested for *C5ar2* with the aforementioned primers, pair 1, 3 and 4. Lanes 1, 2, 3, 5, 6 and 9 were loaded with DNA extracted from C5aR1 KO mice. Lanes 4 and 8 were loaded with extracts from WT mice. Lane 7 corresponds to water control. M1 corresponds to the markers loaded for pair 3 and M2 corresponds to the markers loaded for pair 4.


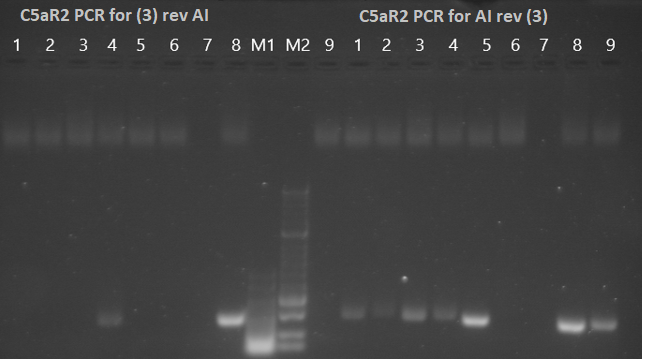


Figure S3: Genotyping C5aR1 KO mice with *C5ar2* primers. C5aR1 KO mice were tested for *C5ar2* with the self-designed primers combined with the AI pairs. A) PCR was performed with tail extracts of WT (lanes 4 and 8), C5aR1 KO mice (lanes 1, 2, 3, 5, 6 and 9) and water control (lane 7) using forward primer from Pair 3 and reverse primer from pair AI. B) PCR was performed with tail extracts of WT (lanes 4 and 8), C5aR1 KO mice (lanes 1, 2, 3, 5, 6 and 9) and water control (lane 7) using forward primer from Pair AI and reverse primer from pair 3. M1 corresponds to the markers loaded for pair 2 and M2 corresponds to the markers loaded for pair 3.

*C5aR2 KO mice genotyping*

C5aR2 KO mice were genotyped with the aforementioned *C5ar1* primer pair and primer pair 1 for *C5ar2*. Apart from the WT mice, all tested C5aR2 KO mice were positive for *C5ar1* (Figure S4. A.) and negative for *C5ar2* (Figure S4. B). Therefore, C5aR2 KO mice were confirmed to have a deficiency of C5aR2 activity and had sufficient C5aR1 activity.


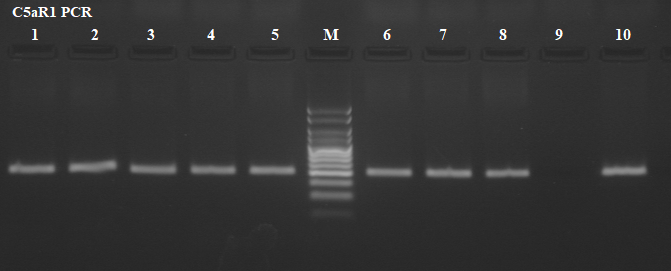
A)


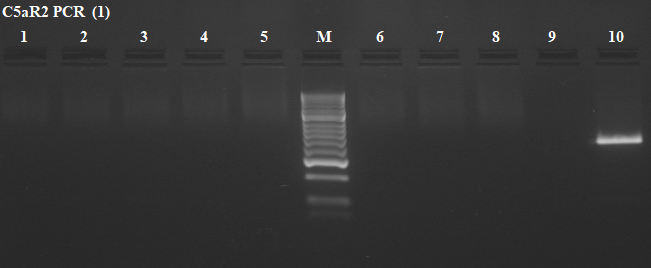
B)

Figure S4: Genotyping C5aR2 KO mice with *C5ar2* primers. C5aR2 KO mice were tested for
A) *C5ar1* with the aforementioned primer pair and B) for *C5ar2* with primer pair 1. Lanes 1–8 correspond to C5aR2 KO, lane 10 corresponds to WT and lane 9 corresponds to water control.

References: -

1. Hopken, U. E., Lu, B., Gerard, N. P. & Gerard, C. The C5a chemoattractant receptor mediates mucosal defence to infection. *Nature* **383**, 86–89 (1996).

2. Gerard, N. P. *et al.* An anti-inflammatory function for the complement anaphylatoxin C5a-binding protein, C5L2. *J. Biol. Chem.* **280**, 39677–39680 (2005).
